# Anti-IL-17A antibody-associated *de novo* vitiligo: Case report and review of literature

**DOI:** 10.3389/fimmu.2022.1077681

**Published:** 2023-01-18

**Authors:** Hsing-Jou Su, Yu-Pei Chan, Peng-Chieh Shen, Cheng-Lung Ku, Chau Yee Ng

**Affiliations:** ^1^ Department of Dermatology, Chang Gung Memorial Hospital, Linkou, Taiwan; ^2^ School of Medicine, College of Medicine, Chang Gung University, Taoyuan, Taiwan; ^3^ Vitiligo Center and Pigment Research Center, Chang Gung Memorial Hospital, Linkou, Taiwan; ^4^ Graduate Institute of Clinical Medical Sciences, Chang Gung University, Taoyuan, Taiwan; ^5^ Department of Physical Medicine and Rehabilitation, Lotung Poh-Ai Hospital, Lo-Hsu Medical Foundation, Inc., Yilan, Taiwan; ^6^ Department of Dermatology, Jen-Ai Hospital, Tai Chung, Taiwan

**Keywords:** vitiligo, anti-IL17 agents, *de novo*, paradoxical reaction, psoriasis, Anti-IL17A-antibody

## Abstract

Interleukin (IL)-17 inhibitor is a biological therapy approved for moderate to severe psoriasis and psoriatic arthritis. The common adverse events of IL-17 inhibitor include injection site reaction, infections, nasopharyngitis, and headache. However, vitiligo associated with the use of IL-17 inhibitors was rarely reported in the previous literature. Here we described a woman who developed *de novo* vitiligo after 4 months of IL-17A inhibitor treatment for psoriasis and psoriatic arthritis. Upon discontinuation of IL-17A inhibitor and shifting to a broader T cell inhibitor—cyclosporine, our patient had control of both psoriasis and vitiligo and achieved 75% repigmentation after 3 months of oral cyclosporine without phototherapy. Due to the increasing use of anti-IL-17 biologics in psoriasis patients, clinicians should inquire about vitiligo’s history before treatment and inform patients of the possible adverse effects.

## Introduction

Interleukin (IL)-17 inhibitor is a biologic therapy approved for moderate to severe psoriasis and psoriatic arthritis (1). The common adverse events of IL-17 inhibitor include injection site reaction, infections, nasopharyngitis, and headache (1). However, vitiligo associated with the use of IL-17 inhibitor was rarely reported in the previous literature (2–4). Here we described a woman who developed vitiligo after IL-17A inhibitor treatment for psoriasis and psoriatic arthritis.

## Case report

**Figure 1:**
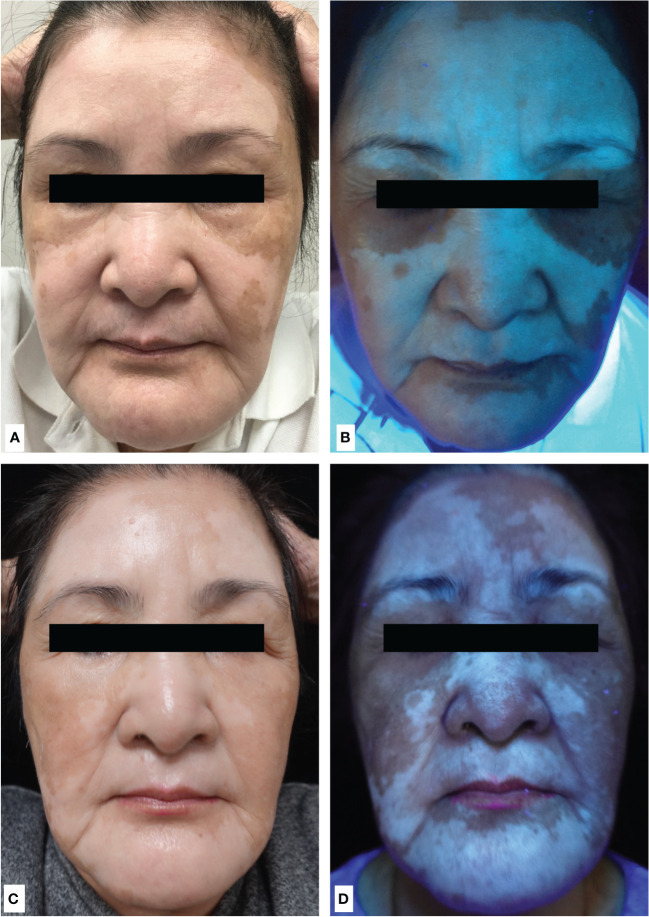
**(A)** Multiple depigmented facial patches sparing the periorbital areas after 4 months of treatment with anti-IL-17A antibody (ixekizumab). **(B)** Wood’s lamp of depigmented patches on the face showed bluish–white fluorescence, confirming the diagnosis of vitiligo. **(C)** Achieving 75% repigmentation of vitiligo under normal light and **(D)** under Wood’s lamp after discontinuation of ixekizumab and 3 months of cyclosporine.

A 72-year-old woman presented with chronic plaque psoriasis and psoriatic arthritis since she was 16 years old. She received methotrexate and TNF-α inhibitors for psoriatic arthritis and psoriasis, but it was unsatisfactory. Her psoriatic plaques cleared, and her arthritis improved under the treatment of an IL-17A inhibitor (ixekizumab). However, she experienced skin erythema, burning sensation, and itchiness in the eighth week of treatment, followed by the rapid development of depigmented patches on the face in the fourth month of treatment (**Figure 1A**). Wood’s lamp examination showed bluish–whitish fluorescence on the depigmented patches, confirming the diagnosis of vitiligo (**Figure 1B**). The laboratory examination for lupus and thyroid profile was unremarkable [anti-nuclear antibody (≤1:80; range: ≤1:80), complement 3 (119 mg/dl; range: 90–180), complement 4 (23.4 mg/dl; degree: 10–40), rheumatic factor IgM (<5 units; range: ≤6), normal anti-thyroid peroxidase antibody (anti-TPO; <3 IU/ml; range: <5.6), normal free T4 (0.95 ng/dl; range: 0.70–1.48), and thyroid-stimulating hormone (1.656 uIU/ml; range: 0.35–4.94)]. We discontinued the IL-17A inhibitor, and her psoriasis worsened gradually with no signs of repigmentation of the face. We treated her with oral methotrexate, topical steroid, and topical calcineurin inhibitor for 2 months, but her response was poor. Subsequently, we shifted to oral cyclosporine (3 mg/kg/day) to control psoriasis and vitiligo. Her psoriasis and arthritis were well controlled under cyclosporine. Interestingly, her facial vitiligo also repigmented and achieved 75% repigmentation in the third month of treatment (**Figures 1C, D**). She did not receive phototherapy during the treatment period due to inconvenience.

## Discussion

Vitiligo is an autoimmune disease with progressive destruction of melanocytes in the skin, resulting in patchy disfiguring depigmentation (5). Recent studies implicate the autoreactive CD8+ T cell-mediated destruction of melanocytes, and IFN-γ is the critical driver of autoimmunity in vitiligo (6). Studies have shown that IFN-γ may contribute to the polarization of T cells to a T helper type 1 (Th1) phenotype (7). Although circulating IL-17 levels and T helper type 17 (Th17) numbers have been shown to increase in vitiligo patients, the pathogenic role of IL-17 and Th17 in vitiligo is debatable (4).

IL-17A inhibitor-associated *de novo* vitiligo is a rare adverse event. Our case developed facial skin redness and itchiness followed by rapid depigmentation after IL-17A inhibitor therapy for chronic plaque psoriasis and psoriatic arthritis. A pilot study using IL-17 inhibitors to treat active non-segmental vitiligo has shown the progression of the disease, thus limiting further enrollment (4). Speeckart et al. found that patients with progression of vitiligo have significantly increased Th17.1 and Th1 lymphocytes, but not Th17 lymphocytes (4). We postulate that targeting IL-17 inhibits Th17, skewing towards a predominantly Th1 response that exacerbates vitiligo. On the contrary, a broad-spectrum T-cell calcineurin inhibitor does not influence the Th1/Th17 balance and therefore controls both psoriasis and vitiligo.

A nationwide cohort study reported a few isolated cases of *de novo* vitiligo following biologic therapy (2). The most commonly reported were TNF-α inhibitors, followed by IL-12/23 inhibitors. However, IL-17A inhibitor was rarely reported. **Table 1** shows a summary of the published cases of IL-17 inhibitor-associated vitiligo in the literature (2–4, 8). There has been primarily reported cases in association with secukinumab (2, 4), one case with ixekizumab (3), and no case with brodalumab. A paradoxical reaction resulting in the appearance or worsening of chronic immune-mediated disease that usually responds to this drug class is not uncommon during biologic therapy (9). A delicate balance in the Th1/Th17 immune response is vital to prevent paradoxical and adverse reactions. Studies have demonstrated that Th1 and Th17 regulate one another through a delicate balance. The lack of one of these corresponding effector cytokines can promote a response dominated by the other. In an animal study, IL-17 deficiency contributes to increased IFN-γ^+^Th1 cells and an elevated Th1 response (10).

**Table 1:** Literature review of anti-IL-17-associated vitiligo in patients with psoriasis.

Biologics	Year, author	Case number	Age, sex	Types	Location	Onset	Treatment	Outcome
Ixekizumab	2021, Su et al. (our case)	1	72, F	*De novo* vitiligo	Face	4 months	Discontinuation of ixekizumab, excimer, and cyclosporine	75% repigmentation of the face after 3 months of cyclosporine with control of psoriasis
	2019, Federico Pirro et al. (3)	1	48, M	*De novo* vitiligo	Legs, hands, feet	3 months	Topical tacrolimus 0.1% ointment for 8 weeks without discontinuation of ixekizumab	50% repigmentation at 4 months of follow-up
Secukinumab	2019, Reinhart Speeckaert et al. (4)	7	Mean: 50, N/A	Exacerbating of preexisting vitiligo	N/A	1–9 months	Discontinuation of secukinumab	N/A
	2016, L. Mery-Bossard et al. (2)	1	N/A	*De novo* vitiligo	N/A	N/A	N/A	N/A

N/A, not available.

Upon the discontinuation of IL-17A inhibitor and shifting to a broader T cell inhibitor—cyclosporine, our patient had control of both psoriasis and vitiligo and managed to achieve 75% repigmentation of facial vitiligo 3 months later without phototherapy. This leaves the question of whether the patient would benefit from a broad-spectrum immunosuppressant or a specific targeted biologic therapy. Considering the balance of Th1/Th17 immune response in patients with concomitant psoriasis and vitiligo, the selection of steroid-sparing immunosuppressants with broader immunosuppressive properties—such as cyclosporine, JAK inhibitors, and methotrexate—may be beneficial (11).

Both vitiligo and psoriasis are common chronic autoimmune skin disorders. Studies have shown that the frequency of vitiligo among psoriasis patients is 2%, and patients with vitiligo showed a 2.29-fold risk of concomitant psoriasis (12). Therefore, a concurrent diagnosis of psoriasis and vitiligo is not uncommon. In contrast to psoriasis, vitiligo is more difficult to treat and irreversible if the melanocyte destruction is too advanced, resulting in permanent “color scarring” that significantly affects the patients’ quality of life and causing social stigma. *De novo* vitiligo or worsening preexisting vitiligo may develop during biologic therapy for inflammatory disorders such as psoriasis. Due to the increasing use of anti-IL-17 biologics in psoriasis patients, when both diseases coexist, clinicians should be cautious when selecting a targeted biologic therapy, and the history of vitiligo should be taken into consideration.

Furthermore, clinicians should inquire about vitiligo’s history before treatment and inform patients of the possible adverse effects. The early recognition of this adverse effect and prompt treatment can also prevent overt melanocyte destruction. Further studies should be conducted for factors predicting the risk of this paradoxical adverse effect. It is worth noting that, due to the complexity of each patient with varied cytokine responses to biologics, immunophenotyping of each patient may be an option to direct personalized biologic therapy.

## Data availability statement

The original contributions presented in the study are included in the article/supplementary material. Further inquiries can be directed to the corresponding author.

## Ethics statement

The studies involving human participants were reviewed and approved by Chang Gung Memorial hospital, IRB no. 202100855B0. The patients/participants provided their written informed consent to participate in this study. Written informed consent was obtained from the individual(s) for the publication of any potentially identifiable images or data included in this article.

## Author contributions

H-JS: manuscript drafting and literature review. Y-PC: literature review. P-CS: literature review. C-LK: Conceptualization. CN: conceptualization, manuscript revision, data acquisition, funding. All authors contributed to the article and approved the submitted version.
